# Beyond weight loss: exploring the neurological ramifications of altered gut microbiota post-bariatric surgery

**DOI:** 10.1186/s12967-025-06201-2

**Published:** 2025-02-24

**Authors:** Rashed T. Almheiri, Baraa Hajjar, Saif M. I. Alkhaaldi, Nadia Rabeh, Sara Aljoudi, Khaled S. Abd-Elrahman, Hamdan Hamdan

**Affiliations:** 1 Department of Biological Sciences, College of Medicine and Health Sciences, Khalifa University, 127788 Abu Dhabi, United Arab Emirates; 2 Department of Anesthesiology, Pharmacology and Therapeutics, and Djavad Mowafaghian Center for Brain Health, University of British Columbia, Vancouver, BC V6T 1Z3 Canada; 3 Department of Medical Sciences, College of Medicine and Health Science, Khalifa University, 127788 Abu Dhabi, United Arab Emirates; 4 Department of Pharmacology and Toxicology, Faculty of Pharmacy, Alexandria University, Alexandria, 21521 Egypt; 5 Healthcare Engineering Innovation Group (HEIG), Khalifa University of Science and Technology, 127788 Abu Dhabi, United Arab Emirates

**Keywords:** Gut-brain-axis, Neurological disorders, Neurodegeneration, Neuropsychiatric, Bariatric surgery, Microbiota, Neurotransmitters, Neuroendocrine

## Abstract

This review discusses findings related to neurological disorders, gut microbiota, and bariatric surgery, focusing on neurotransmitters, neuroendocrine, the pathophysiology of bacteria contributing to disorders, and possible therapeutic interventions. Research on neurotransmitters suggests that their levels are heavily influenced by gut microbiota, which may link them to neurological disorders such as Alzheimer's disease, Parkinson's disease, Multiple sclerosis, Depression, and Autism spectrum disorder. The pathophysiology of bacteria that reach and influence the central nervous system has been documented. Trends in microbiota are often observed in specific neurological disorders, with a prominence of pro-inflammatory bacteria and a reduction in anti-inflammatory types. Furthermore, bariatric surgery has been shown to alter microbiota profiles similar to those observed in neurological disorders. Therapeutic interventions, including fecal microbiota transplants and probiotics, have shown potential to alleviate neurological symptoms. We suggest a framework for future studies that integrates knowledge from diverse research areas, employs rigorous methodologies, and includes long-trial clinical control groups.

## Introduction

The awe-inspiring complexity of the human gastrointestinal tract is characterized by its intricate role in digestion, absorption, and microbial processes. These processes rely on a dynamic interplay between the body and the external environment, significantly influencing the variety of resident microorganisms expressed in the gut. Accumulating evidence highlights that external factors altering the gut microbiota result in changes to microbial metabolites and, consequently, physiological levels of homeostasis [1]. Such changes can have deleterious effects on major organ systems, namely the nervous system and its bidirectional communication with the gut. A disruption in the gut-brain axis leads to physiological disturbances that can accelerate the progression of neurological illness. Neurological conditions associated with gut-brain axis disturbances include Alzheimer’s disease (AD), Parkinson’s disease (PD), Multiple sclerosis (MS), Depression, and Autism spectrum disorder (ASD) [2]. Elucidating the relationship between the gut microbiota and neurological disorders can aid in developing therapeutic strategies.

Of the countless external factors altering the gut microbiota, bariatric surgery’s effects on treating obesity have been understudied. As a global health concern, obesity has been associated with changes in the gut microbiota [3]. A marked imbalance in the abundance and diversity of gut microorganisms is observed in obese patients. This dysbiosis is a series of metabolic and inflammatory derangements resulting in additional comorbidities such as type 2 diabetes mellitus (T2DM) [3, 4]. Several treatment options, ranging from conservative or medical treatments to surgical procedures, have been employed in managing obesity. Surgical interventions are used last when all non-surgical treatments have failed or are offered to individuals with a body mass index (BMI) greater than or equal to 35 kg/m^2^ [5]. Such interventions either resect or bypass parts of the intestines, leading to drastic weight loss in people living with obesity. Common bariatric surgeries include gastric bypass, Roux-en-Y gastric bypass (RYGB), and sleeve gastrectomy (SG). In RYGB, a small pouch is made from the proximal portion of the stomach and is connected to the jejunum, bypassing the remaining stomach and duodenum. SG removes a significant portion of the stomach without bypassing structures [6]. Evidence shows that such surgeries significantly impact the gut microbiota [7]. However, whether these changes positively or negatively affect the gut-brain axis and the progression of neurological conditions remains unclear. In this review, the changes to the gut microbiome following bariatric surgery will be explored in relation to its effect on the gut-brain axis and in the development of neurological conditions.

## Gut microbiota composition and diversity

The gut hosts over 100 trillion microorganisms, with bacteria being the primary microorganism and a small proportion of viruses and yeast [3, 8]. Bacteria are classified based on their phylum, class, order, family, genus, and species. The two most prevalent phyla in human adult gut microbiota are Firmicutes and Bacteroidetes, in contrast to the less abundant Actinobacteria, Proteobacteria, and Verrucomicrobia [9]. Within the Firmicutes phyla are various genera, with the prominent ones being *Streptococcus, Enterococcus,* and *lactobacillus* [10]. In the Bacteroidetes phyla, *Prevotella* and *Bacteroides* are the most abundant genera [10].

A universal standard composition of microorganisms in healthy human guts does not exist [11]. The gut microbiota changes relative to an individual’s development, such as age, form of delivery, diet, BMI, and gastrointestinal region [11]. The external environment, including a person’s geographical location, ethnicity, and the local climate, can also significantly influence the gut microbiota [11]. In individuals with obesity, the abundance of Firmicutes decreases, while that of Bacteroidetes increases. [12]. Within the Bacteroidetes phyla, patients living with obesity host a higher concentration of the *Prevotella* genera compared to healthy, control patients [12]. Variability within the gut microbiome of people with obesity has been attributed to metabolic status, an indicator dependent on age, weight, and cholesterol levels. Compared to the metabolically healthy patients, metabolically unhealthy patients had a higher proportion of Fusobacteria [13]. The microbiota of metabolically healthy individuals with obesity were enriched with the *Clostridium* genus [13]. Differences in gut microbiota can be due to a constellation of factors, highlighting the increasingly personalized nature of the gut microbiome and its implication in diseases.

Usually, persisting post-operative microbiota alterations occur in response to physiologic and anatomic changes. Examples of driving forces inducing changes to the gut microbiota are decreased acid production, changes to the concentration of bile acid reaching the colon, and elevated oxygen content [14]. Additional factors dictating the surgery’s success and post-operative changes to the gut microbiome include pre-surgical microbiome composition, diabetes remission, dietary changes, and inflammation [15–18]. These changes can be stratified based on the type of surgical procedure. RYGB and SG have been the two most common procedures studied in the context of post-operative changes to the gut microbiota. There is a consensus that either procedure leads to an increase in the abundance of Proteobacteria. Within the Proteobacteria, both procedures increased *Escherichia coli* [19]. An increase in Proteobacteria is associated with inflammation and disease [20]. Findings regarding the abundance of the Firmicutes phylum are contradictory, with some papers citing a decrease or no change in abundance [21, 22]. However, the majority of studies cite a reduction in Firmicutes. Another common genus seen to increase after RYGB and SG is *Streptococcus* [23, 24]. The changes in gut microbiota after surgery also seemed to depend on the patient’s diabetic status. In RYGB and SG, *Roseburia intestinalis* increased in patients achieving diabetes remission, while individuals with persistent diabetes had elevated pre-operative levels of *Desulfovibrio* species [18, 19]. Research investigating the post-operative alterations to the gut microbiota is invaluable as it enriches our understanding of successful or unsuccessful surgical outcomes.

The anatomical changes occurring during RYGB cause a physiological change that increases the abundance of certain microorganisms, including *Klebsiella*, *Escherichia*, and *Pseudomonas* facultative anaerobes [22]. Some instances of RYGB show an increase in the *Akkermansia* genus, part of the Verrucomicrobia phylum, which is associated with improved metabolism [25, 26]. There is a lack of consensus about the post-operative abundance of *Blautia* species, with studies citing an increase or decrease in abundance [25, 26]. A reduction in *Clostridium hiranonis* and *Clostridium difficile* from the Firmicutes phylum, as well as the *Bifidobacterium* genus from the Actinobacteria phylum, was noted after RYGB [22]. There is debate regarding the post-operative change in *Faecalibacterium prausnitzii* and *Coproccocus* species [19]. However, these differences could be attributed to patient diets, sample acquisition and processing, demographics, and environments [19]. In SG, *Faecalibacterium prausnitzii*, *Roseburia intestinalis*, and *Bulleidia* species from the Firmicutes phylum tended to increase, which was also seen in the Bacteroidetes phyla [19]. For example, an abundance and loss of Actinobacteriota and Proteobacteria, respectively, were only reported nine months after SG [23]. A reduction in *Butyricicoccus*, *Agathobacter*, *Lachnospiraceae UCG-010*, and *Eubacterium hallii* also occurred nine months after SG [23]. It is important to note that changes to the gut microbiota are not permanent or static. The dynamic state of the gut microbiome is seen post-operatively when the concentration of certain microorganisms fluctuates. For example, following RYGB, Verrucomicrobia, and Proteobacteria concentration changes diminish after 12 months [21]. Moreover, a study has shown a significant augmentation in *Escherichia*, *Klebsiella*, and *Pseudomonas* 9 years after gastric bypass surgery [27]. A summarized list of selected microbes from the latest reviews of post-bariatric microbiota changes can be seen in Table 1 [19, 23, 27–33].

**Table 1:** Changes in bacterial composition post-bariatric surgery

Bacteria	RYGB	SG	Source
Escherichia (Proteobacteria)	↑	–	[29]
Gammaproteobacteria	↑	–	[32, 33]
Klebsiella (Proteobacteria)	↑	↑	[27, 29]
Akkermansia (Verrucomicrobia)	↑	↑	[28, 29]
Faecalibacterium prausnitzii (Firmicutes)	↓	↑	[19]
Shigella	↑	–	[29]
Enterococcus faecalis	↑	–	[30]
Pseudomonas (Proteobacteria)	↑	–	[30]
Roseburia	↑↓	↑	[30]
Clostridium species	↑↓	↑	[30]
Coprococcus comes	↓	↓	[27, 29]
Adlercreutzia	–	↓	[31]
Lactobacillus (Firmicutes)	↓	–	[29]
Bifidobacterium (Actinobacteria)	↓	↓	[27, 29]
Proteobacteria	↑	–	[27, 30]
Enterobacteriaceae	↑	–	[30]
Enterococcus faecalis	↑	–	[30]
Butyricicoccus	-	↓	[23]
Lachnospiraceae	-	↓	[23]
Blautia	↓	↑	[28, 30]

The association between bariatric surgery (RYGB or SG) and bacteria. The sources are selected from the latest reviews

The gut microbiota has also been shown to play a fundamental role in modulating the overall immune system and in inflammation, particularly neuroinflammation [34]. The release of metabolites mediates the crosstalk between the immune system and gut microbiome. Depending on the gut microbiome’s diversity, various metabolites are released and have been implicated in cases of neurodegenerative diseases or neurological conditions. For example, short-chain fatty acids (SCFA) released by bacteria of the *Clostridium*, *Roseburia*, or *Akkermansia* genera can maintain the integrity of the gut barrier as well as the blood–brain barrier (BBB) [34]. However, in studies on mice modeling AD, SCFAs were found to promote the accumulation of amyloid β (Aβ) plaques in the brain. Fecal SCFAs were also lower in patients with PD [34]. Tryptophan metabolites are additional metabolites that have been implicated in disease development. Originally, tryptophan metabolites released from bacteria of the *Clostridium*, *Lactobacillus*, or *Pseudomonas* genera play a role in neurogenesis, as well as neuronal proliferation and development [34]. Indole, a tryptophan metabolite, has been shown to promote development of anxiety and mood disorders, play a role in accumulation of pathogens in the gut, and disrupting the gut-brain axis in patients with ASD [35]. Interestingly, a rodent experiment also suggested increased microbiota-derived indole post-bariatric surgery [36]. This suggests that bariatric surgery can influence the levels of indole, potentially disrupting the gut-brain axis. These studies highlight the essential interaction between the nervous system and the gut microbiota composition, critical for comprehending and addressing neurological conditions.

## Potential mechanisms of gut microbiota modulation by gastric bypass

Gastric bypass surgeries result in metabolic imbalances, including macronutrient and micronutrient deficiencies [37, 38]. Various mechanisms and theories explain these effects, spanning from alterations in anatomy to changes in gut microbiota. This section examines the mechanisms through which gastric bypass surgeries modulate the gut microbiota.

### Glucagon-like peptide 1 & peptide YY

The interaction between gut microbiota and gut hormonal secretions is bidirectional, as bacteria respond to these hormones while also producing or metabolizing them [39]. Therefore, changes in hormonal secretions following bariatric surgery are expected to affect the gut microbiota composition.

Additionally, the gut microbiome feeds on the body's ingested material. However, if there are any anatomic alterations, there will be changes in absorption time, and nutrient flow pattern [40]. Therefore, nutrient delivery to microbes will vary based on the new alterations, as certain places will not receive nutrients as their presurgical patterns. This will affect the composition of gut microbes that feed on these nutrients, and subsequently, the type and levels of hormones they produce as well [40].

Hormones demonstrate a spatially dependent release, where, for instance, glucose-dependent insulinotropic polypeptide (GIP) is secreted primarily from K cells in the duodenum, whereas glucagon-like peptide 1 (GLP-1) is released mainly from L cells in the distal parts of the small intestine [41]. GIP and GLP-1 are incretin hormones that stimulate insulin secretion in response to glucose ingestion [42]. In structural alterations involving the duodenum, such as RYGB, the secretion of GIP is expected to be altered, usually decreased [43]. In contrast, the secretion of GLP-1 is likely to increase as food is being routed directly to the distal part of the small intestine, thereby overstimulating these areas and resulting in more significant hormonal secretions from cells in these specific areas [44]. A cross-sectional study comparing RYGB patients with controls observed a markedly increased postprandial insulin in gastric bypass patients [45]. This effect may be attributed to the post-surgical increased activity of GLP-1[46, 47]. The use of a GLP-1 receptor blocker corrected postprandial hypoglycemia in patients who underwent gastric bypass, indicating that GIP does not play a major role in the mechanism of hypoglycemia, which instead appears to be driven primarily by the increased GLP-1 activity [47]. This outcome is thought to be due to the alterations in the anatomy of the gastrointestinal tract, routing the food directly into the intestines, reducing gut transit time, and thereby resulting in increased hormonal secretion from the respective portions of the intestines that experience greater nutrient flow rate [44]. Interestingly, GLP-1 levels post-surgery have been elevated even after 10 years, highlighting the sustained improvement in GLP-1 release following surgery [48]. Moreover, the impact of GLP-1 on insulin levels may have a neuroprotective effect against neurodegenerative diseases [49]. For example, GLP-1 analogs are under investigation for potential neuroprotective roles in Alzheimer's disease (AD) [50].

Moreover, alterations affecting the transit time while maintaining passage through the duodenum, such as in SG, also produce similar effects as food spends less time in the stomach, and absorption occurs more in distal parts, resulting in an exaggerated hormonal secretion distally [44]. In addition to GLP-1, peptide YY (PYY) is another hormone produced distally in the small intestine by L-cells, stimulating satiety and suppressing appetite [51]. Hormones like GLP-1 and PYY are, therefore, more elevated postprandially in bariatric surgery patients than in healthy controls; this elevation is more profound in RYGB than in SG [52]. Elevated PYY plays a potential role in affecting mood, anxiety, and cognition through the Y receptor family [53]. Levels of other hormones, including leptin, adiponectin, and ghrelin, also change after bariatric surgery [40].

### Ghrelin and adipokines

Diet affects the gut microbiota through modulating appetite and food intake [54]. For example, ghrelin and leptin levels are altered post-bariatric surgery, and these two hormones manage hunger and satiety, respectively [55]. Therefore, changes in their levels will affect food intake, impacting the gut microbiota composition.

Ghrelin exhibits complex and sometimes conflicting patterns following bariatric surgery [40]. Notably, *Faecalibacterium*, a SCFA-producing organism, has been shown to downregulate ghrelin production [56]. Moreover, a rodent study suggests that ghrelin may play a role in modulating inflammation, as its levels can be influenced by inflammatory cytokines triggered by lipopolysaccharide (LPS) in response to *Helicobacter pylori* [57]. Together, these findings suggest that both inflammatory cytokines and SCFAs play an active role in regulating ghrelin release. Interestingly, ghrelin has been shown to have neuroprotective effects. Individuals suffering from Parkinson’s disease (PD) and AD exhibit disrupted ghrelin release [58, 59]. Moreover, ghrelin plays a role in memory, mood, and other brain functions [58, 59]. Thus, ghrelin release may have a positive effect on the brain and changes may be a sign of neurological disorder progression.

Adipokines, which include Leptin and Adiponectin, are a group of hormones associated with satiety. Recent research on cell lines and rat models has revealed that certain adipokines, particularly leptin, show extraordinary functions such as neuroprotective effects [60, 61]. Moreover, leptin is a gut hormone that is greatly altered post-bariatric surgery and shown to be decreased post-RYGB [40, 62]. Lower levels of leptin are associated with neurodegenerative and neuropsychiatric disorders [61]. Similarly, adiponectin is another hormone influenced post-bariatric surgery and was shown to be increased [40, 62]. Changes in the levels of adiponectin may as well have neurological manifestations, given the expression of adiponectin receptors in the brain [63]. For instance, low levels of adiponectin and increased leptin resistance, as in obesity, were found to increase the risk for neurodegenerative diseases, which makes these hormones potential targets for neurodegenerative diseases like AD [64]. This elucidates some of the gut hormonal mechanisms underlying the neurological consequences of bariatric surgeries.

### Bile acid metabolism alterations

The interaction between gut microbiota and bile acids represents another crucial aspect of the gastrointestinal system. Primary bile acids are made in the liver and the gut microbiota metabolizes it forming secondary bile acids, bile acids have protective functions and exert control over the gut microbiota [65–67]. Bile acids were increased in patients who underwent RYGB compared to controls with matched body mass index (BMI) [68]. Contrary to the RYGB, SG surgeries resulted in little or no significant differences in bile acids post-surgery [69]. The change in bile acid concentrations following bariatric surgery may be due to the anatomical alterations of bile acid drainage and modifications of the enterohepatic circulation, especially in RYGB [70].

Moreover, bile acids serve signaling roles that, in turn, influence the gut microbiota [70]. For instance, bile acid receptors, known as Takeda G Protein-Coupled Receptor 5 (TGR5), are found in both the brain and the gut. These receptors promote GLP-1 release and have been shown to reduce inflammation [71]. Remarkably, a rat model of gastric bypass surgery was utilized to analyze the metabolite profile, revealing a reduction in secondary bile acids metabolized by gut bacteria in the gastrointestinal tract [72]. This suggests a decrease in the population of bacteria responsible for generating secondary bile acids after bariatric surgery, leading to reduced stimulation of TGR5. Since TGR5 activation suppresses inflammatory cytokines, a reduction in secondary bile acids and subsequent TGR5 activity may contribute to increased systemic inflammation [73]. Studies have shown that bile acids, particularly hydrophilic ones, possess neuroprotective potential, including regulating apoptotic pathways, reducing oxidative stress, protecting mitochondria, and exerting anti-neuroinflammatory effects. Numerous preclinical animal studies have demonstrated these neuroprotective pathways in AD and PD [74]. Interestingly, Tauroursodeoxycholic acid (TUDCA), a hydrophilic secondary bile acid, was shown to significantly influence and reduce amyloid β deposition in AD mice models [74]. Similarly, in a PD mouse model, TUDCA treatment has been associated with mild to absent motor symptoms [75]. Lastly, in MS mice models, altered bile acid metabolism has been observed, accompanied by reduced neuroinflammation [76]. However, studies on mice have shown an increase in TUDCA serum levels in vertical SG [77]. Moreover, a study conducted on patients with obesity post-bariatric surgery has shown a decrease in TGR5 gene expression, while a study conducted on mice demonstrated a reduction in secondary bile acid levels in ileal biopsies & luminal samples of different sections of the gut following RYGB [72, 78, 79]. These mixed results warrant a thorough investigation into the effects of bile acids in relation to neurodegeneration and bariatric surgery.

### Short chain fatty acids metabolism alterations

SCFA, metabolites produced by gut microbiota, play key roles by stimulating enteroendocrine cells such as increasing GLP-1 and PYY release [52]. Their levels can be expected to change after bariatric surgery due to alterations in gut microbiota composition. Moreover, dietary changes following bariatric surgery are another factor that may affect SCFA levels, as these are fermentation products of dietary fiber [80]. For instance, a cohort study observed decreases in fecal levels of SCFAs, particularly propionate, butyrate, valerate, acetate, and other straight SCFAs, following RYGB and SG [80]. In contrast, another study reported increased plasma levels of butyrate and propionate in RYGB patients post-surgery while decreases in other plasma SCFAs, including acetate and valerate, were noted [81]. These differences suggest that certain SCFAs, such as butyrate and propionate, may be more readily absorbed from the gut into the bloodstream due to the anatomical changes caused by the surgery, as evidenced by their decreased fecal levels and increased plasma levels. In contrast, other SCFAs, such as acetate and valerate, appear to be absolutely decreased, possibly due to alterations in microbiota composition, with reductions observed in both fecal and plasma levels post-surgery [80, 81].

Further analysis of fecal samples showed that straight-chain SCFAs tend to decrease after surgical intervention, while branched-chain SCFAs, such as isobutyric acid, isovaleric acid, and isocaproic acid, show a less pronounced increase, resulting in an overall reduction in total SCFAs [80, 82]. The decrease in straight SCFAs and the mild increase in branched SCFAs indicate an unfavorable shift from a predominantly saccharolytic type of pattern to a more proteolytic type of fermentation, which is generally associated with worse health outcomes [80, 82]. Moreover, SCFAs, particularly straight SFCAs, are well known to possess anti-inflammatory properties, and therefore, an overall decrease in their levels further contributes to long-term adverse health effects [83].

Overall, SCFAs were shown to have neurological effects and were associated with neurodegenerative diseases, including AD and PD, highlighting the need for further investigation [84]. These topics show how deeply intertwined the gut-brain axis is and how simple modulation of the gut anatomy can drastically impact the normal gut microbiota through a variety of mechanisms.

## Overview of the gut-brain axis

**Fig. 1:**
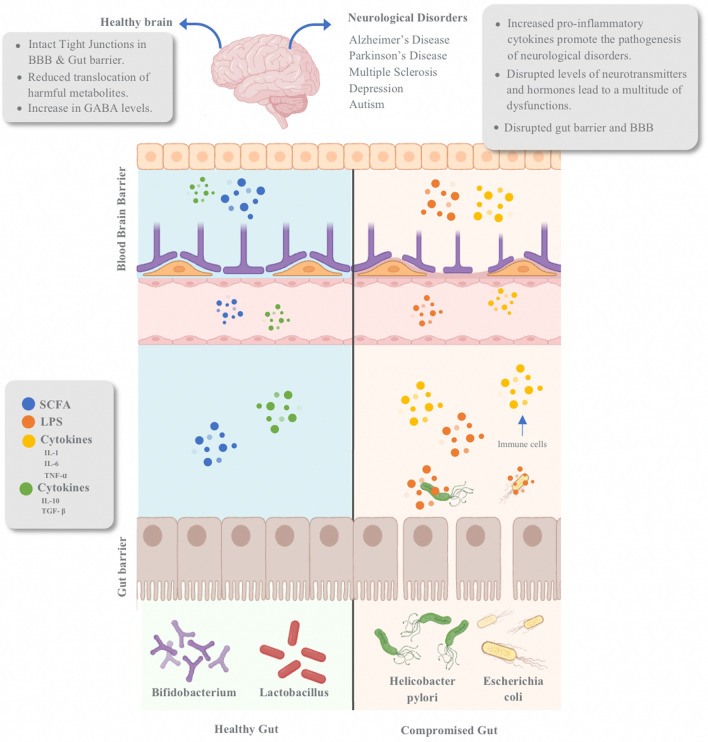
Schematic diagram representing the gut-brain axis and potential consequences. The gut-brain axis is a crucial link between the gastrointestinal tract and the brain. In a healthy gut, beneficial microbes such as Bifidobacterium and Lactobacillus promote anti-inflammatory responses by releasing cytokines that suppress inflammation. This action can reduce the potential effects of neuroinflammation. Conversely, harmful microbes like Helicobacter pylori and Escherichia coli exacerbate inflammation in a compromised gut. They do this by releasing pro-inflammatory cytokines that intensify inflammation and weaken the connections between the gut wall cells, facilitating the translocation of harmful substances. This dynamic illustrates the significant impact of gut microbiota on overall brain health and inflammation pathways. This diagram was adapted from Liang et al. and L. Liu, Huh, et al. [2, 89]. *SCFA* short-chain fatty acid, *LPS* Lipopolysaccharide

The widely accepted concept that recognizes the gut as the body’s ‘‘second brain’’ is well-established. Both the central nervous system (CNS) and the enteric nervous system in the gut exhibit a remarkable density of neurons and neurohormone production [2, 85]. The gut-brain axis constitutes a dynamic interaction between the CNS, the gastrointestinal system, and the gut's microbial inhabitants, enabling bidirectional communication and influencing each other [86]. This communication is facilitated via three main pathways: neuronal, endocrine, and immune [87]. Gut microbiota can modulate receptor activities, neurotransmission, and metabolite entry into the brain using the aforementioned pathways, potentially altering neuronal plasticity, memory formation, and motivation [88]. The gut microbiota, crucial components of the gut-brain axis, release vital metabolites and neurotransmitters like SCFAs, serotonin (5-HT), and Gamma-aminobutyric Acid (GABA) [88]. Nonetheless, dysbiosis, an imbalance in the gut microbiota, can disrupt this communication and is associated with the onset of neurodegenerative conditions, such as AD, and neuropsychiatric disorders like Depression [2]. Indeed, neuroinflammation is possibly attributed to alterations in the permeability of both the gut barrier and the BBB [88, 89]. A summarized diagram of the gut-brain axis is outlined in Fig. 1.

### Neuronal pathway

The gut microbes can directly influence the brain through the neuronal pathway by synthesizing or breaking down neurohormones, including 5-HT, GABA, Dopamine, Acetylcholine (ACh), and Glutamate to communicate with the CNS [90]. Signals generated by these neurotransmitters in the gut are transmitted to the brain via afferent vagus nerve fibers, and the brain responds to these signals through efferent vagus nerve fibers to the enterochromaffin cells and enteroendocrine cells in the gut [90].

For example, a *Lactobacillus* strain has been shown to have an antidepressant effect. Interestingly, vagotomized mice—mice who do not have a vagus nerve—did not exhibit those behavioral effects even after *Lactobacillus* ingestion, suggesting the importance of vagus nerves in the connection between the gut and the brain [91]. The role of the sympathetic nervous system, whose pathway is still unclear, has been shown to affect gut barrier permeability [88]. Furthermore, the composition of the microbiota has been found to promote neurogenesis. It plays a crucial role in the early-life development of the brain and the apoptosis of neurons [88].

### Endocrine pathway

Microbes can also influence the endocrine glands, impacting the concentration and function of endocrine hormones released in the body. This is believed to be due to several mechanisms which include components such as the vagus nerve, neuropeptides, SCFA, opioid peptides, and neurohormones [88]. As the microbiota metabolites stimulate the brain through mechanisms such as influencing neuroinflammation, synthesizing neurotransmitters, or other processes discussed in later sections in this review, the brain sends signals to various glands, thus indirectly affecting the endocrine system through four main neuroendocrine axes.

Firstly, the Hypothalamus Pituitary Gonad (HPG) axis plays a role in producing luteinizing hormone (LH) and follicle-stimulating hormone (FSH), which subsequently interact with the gonads, prompting the release of estrogen and testosterone [92]. An observational study in humans showed that fecal levels of certain *Coprococcus* species, specifically *Coprococcus_2*, was increased in individuals with obesity and Polycystic Ovary Syndrome (PCOS), a condition where the LH/FSH ratio is disrupted [93]. This could potentially be an important factor that contributes to the ratio alteration that occurs in PCOS by influencing the HPG axis. Moreover, PCOS shares common risk factors with AD, such as insulin resistance [94]. Additionally, depression has been suggested as a potential factor in developing PCOS [95]. Interestingly, low fecal levels of *Coprococcus* in humans were also associated with depression [96].

Secondly, the Hypothalamus Pituitary Adrenal (HPA) axis affects the adrenal cortex, resulting in changes in cortisol levels in response to stress. The HPA axis also regulates digestion, mood, emotions, sexuality, energy expenditure, immunity gain, and the development of the nervous system [97]. Interestingly, germ-free mice have been shown to have exaggerated HPA response and reduced sensitivity to negative feedback which normally maintains cortisol within the normal range. This response was reversed with the addition of *Bifidobacterium* and exacerbated when *Escherichia* was added [88]. Moreover, a study using fecal microbiota transplant (FMT) on germ-free mice has shown that only early-life recolonization of bacteria restores HPA axis function, whereas later recolonization does not, highlighting the crucial role of bacteria in the maturation of the HPA axis [97]. This indicates that the lack of a developed HPA axis or early gut colonization by *Escherichia* can potentially exaggerate the HPA axis, impairing negative feedback and leading to an overall increase in cortisol, which was shown to be associated with neurodegenerative diseases, particularly AD [98].

Thirdly, the Hypothalamic–neurohypophyseal axis (HN) releases oxytocin, which is essential in the stimulation of milk ejection and uterine contractions, and it is involved in social behaviors such as social recognition, pair bonding, maternal behaviors, and managing stress. Some studies have shown that injecting oxytocin into patients can reduce stress levels [97]. Moreover, it’s shown that depressed patients tend to have lower oxytocin levels [99]. Interestingly, a strain of *Lactobacillus* has been shown to upregulate oxytocin levels [100]. As discussed earlier, vagotomized mice have also been shown to reduce the beneficial effect of Lactobacillus [100].

Lastly, the Hypothalamus Pituitary Thyroid (HPT) axis influences thyroid hormone levels, which is involved in essential regulators of metabolism and has various effects, such as regulating appetite, cholesterol secretion into bile, heart contractility, and brain development [97]. Both hypothyroidism and hyperthyroidism were often associated with reduced *Lactobacillus* and *Bifidobacterium*. Moreover, the Supplementation of *Lactobacillus* has been shown to have an improvement in thyroid function [101]. Interestingly, hypothyroidism is associated with early-onset AD and PD [102, 103].

These endocrinal axes show the importance of gut microbiota and its effect on endocrine glands, and disturbances of these glands are associated with neurological disorders. Interestingly, the majority of the microbiota mentioned in this section that promotes the disturbance of the endocrine function overlaps with the gut microbiota changes of post-bariatric surgery, as shown in Table 1. Thus, additional studies are warranted to thoroughly investigate the prolonged impact of changes in endocrine function resulting from bariatric surgery.

### Immune pathway

The immune pathway is established by commensal microbes during birth, facilitating the development of innate immunity (e.g., macrophages and dendritic cells) and adaptive immunity (e.g., B cells and T cells), as well as microglial cell development [104, 105]. This differentiation between beneficial immunity, active against pathogens, and harmful immunity, active against commensal microbes, occurs early in life, encompassing both innate and adaptive aspects [106, 107].

In innate immunity, the expression of Toll-like receptors (TLR) is observed in enterocytes, and its overactivation can lead to inflammation [108]. Notably, excessive activation of TLR-4 in the peripheral system has been linked to stress-related mental disorders and substance abuse [109, 110]. In adaptive immunity, the microbiota is involved in antibody production during early life. Furthermore, the generation of immunoglobulins, like IgA, is crucial in fostering an anti-inflammatory response [111]. However, it has been demonstrated that the absence of microbiota before the first colonization during infancy leads to a decrease in IgA and an increase in IgE, rendering individuals more susceptible to infections [111]. Furthermore, the composition of the gut microbiota can influence whether naive CD4 T cells differentiate into a pro-inflammatory (Th1 and Th17) or anti-inflammatory response (Th2 cells and regulatory T cells) [88]. The absence of microbes has been shown to promote naive T cells to differentiate into Th17 cells (pro-inflammatory), thereby contributing to the pathogenesis of various autoimmune diseases [88, 112]. In both types of immunity, the integrity of the gut barrier relies on the gut microbiota. The absence of microbiota results in a more inflamed barrier and, consequently, higher gut permeability and BBB [113, 114]. Tight junctions (TJs), essential for maintaining the barrier and regulating permeability between the gut and the body, can become compromised during inflammation. This loosening of TJs between epithelial cells permits the translocation of bacteria and cytokines, which induces systemic inflammation propagating it to the BBB, affecting its permeability and function [88, 115].

This implies that early colonization of bacteria is an essential factor in determining the type of response the body will have and if the colonization of the gut could lead to inflammation due to increased expression of TLRs, reduced level of IgA, and increased formation of pro-inflammatory immune cells. This inflammation can have downstream effects on the brain, potentially contributing to the development of neurodegenerative disorders.

### Role of gut microbiota in neurotransmitter production

In the preceding section, it was clarified that the microbiota serves as a crucial contributor to the synthesis and breakdown of various neurotransmitters, each capable of eliciting excitatory or inhibitory effects. This section will further elaborate on each neurotransmitter, starting with serotonin (5-HT). Notably, about 90% of the body’s 5-HT is produced in the gut by enterochromaffin cells using the enzyme Trp Hydroxylase 1 (TpH1). The remaining 10% might be synthesized in the brain through the enzymatic activity of Trp hydroxylase 2 (TpH2) or directly by microbial synthesis [116]. The biosynthesis of periphery 5-HT begins with the conversion of tryptophan, found in the protein diet, to 5-hydroxytryptophan (5-HTP) and culminates in the formation of 5-HT within enterochromaffin cells, mediated by the enzymatic action TpH1 [117].

Furthermore, secondary bile acids and SCFA upregulate the formation of the enzyme TpH1, further facilitating the conversion to 5-HT [118]. It is noteworthy that while 5-HT is typically unable to traverse the BBB, its precursor, 5-HTP, possesses this capability [117]. This means that the brain can produce 5-HT using 5-HTP precursors from the gut. Specific gut microbes, including *Clostridial* species and *Staphylococcus*, have been identified as critical contributors to 5-HT synthesis [119]. *Clostridium* species possess 7α-dehydroxylation which is an enzyme that produces deoxycholate (secondary bile acid) from cholate (primary bile acid), and as deoxycholate interacts with enterochromaffin cells, it upregulates TpH1 synthesis, resulting in more peripheral production of 5-HT [116]. *Staphylococcus*, via the enzyme staphylococcal aromatic amino acid decarboxylase (SadA), converts 5-HTP to 5-HT [120]. This explains the importance of gut microbiota in generating 5-HT either by upregulating TpH1 or directly producing 5-HT. However, associations were made between neurological disorders and high levels of *Clostridium* species, overlapping with the post-bariatric surgery gut microbiota trend, as seen in Table 1 [121–123]. Researchers conducting a study on an AD mouse model found that increases in Clostridium species are associated with elevated levels of deoxycytidine, a metabolite that contributes to the dysregulation of the pyrimidine metabolic pathway. This association suggests that such microbial and metabolic changes may be potential risk factors for AD [124]. Moreover, as discussed in Sect. ‘‘Bile Acid Metabolism Alterations’’, secondary bile acids are reduced post-bariatric surgery, reducing the formation of TpH1.

Dopamine, classified as both an excitatory and an inhibitory neurotransmitter, is synthesized within dopaminergic neurons using tyrosine and L-DOPA as precursors [125]. Interestingly, more than 50% of Dopamine is synthesized in the gut and can be explained by the activity of the SadA enzyme, which converts L-DOPA to Dopamine, which is present in *Staphylococcus* [119]. There are no reports of association within post-bariatric surgery regarding the levels of *Staphylococcus*. However, low levels of dopamine were associated with PD and depression [126, 127].

ACh, a well-recognized neurotransmitter with excitatory properties, is generated through the catalytic function in central nervous system neurons by the enzyme choline acetyltransferase, utilizing choline and acetyl coenzyme A as precursor molecules [119]. Choline is present in the diet; however, certain microbes, including *Corynebacterium* and *Arcanobacterium*, can hydrolyze sphingomyelin to choline using the enzyme phospholipase D [128]. It is important to note that ACh itself cannot traverse the BBB; however, its precursor, choline, can transverse this barrier through specific carriers located on brain microvascular endothelial cells. Critical gut microbiota involved in the synthesis of ACh encompasses *Lactobacillus plantarum*, *Bacillus acetylcholini*, *Bacillus subtili*s, *Escherichia coli*, and *Staphylococcus aureus* [119]. It is believed that a low level of ACh is associated with AD, which is why cholinesterase inhibitors are used to help alleviate certain symptoms of AD [129]. In Table 1 and Table 2, low levels of *Lactobacillus* were associated with AD, and it resembles the gut microbiota profile of post-bariatric surgery. Moreover, *Escherichia* levels were shown to be increased in patients with AD and post-bariatric surgery as well.

**Table 2:** Changes in gut microbiota in neurological disorders

Condition	Bacteria	Result	
		Increase	Decrease
Alzheimer disease	Pseudomonas	[159–161]	–
	Escherichia	[163–166]	–
	Shigella	[163, 165, 166]	–
	B. thetaiotaomicron	[136]	–
	Bifidobacterium	–	[168–170]
	Lactobacillus	–	[170]
	Coprococcus	–	[163]
	Akkermansia	[200]	[176, 200]
Parkinson’s Disease	Proteobacteria	[180, 182, 183]	–
	Enterobacteriaceae	[181–183]	–
	Klebsiella	[187, 199]	–
	Coprococcus	–	[180]
	Blautia	–	[180, 190]
	Roseburia	–	[180]
	Bifidobacterium	[189, 190, 195]	–
	Gammaproteobacteria	[198, 199]	–
	Akkermansia	[200]	–
Multiple sclerosis	Faecalibacterium prausnitzii	–	[205, 211]
	Bifidobacterium	–	[206]
	Coprococcus	–	[206]
	Lachnospiraceae	–	[206]
	Butyricicoccus	–	[206]
	Akkermansia	[122, 200, 205, 211]	–
	Pseudomonas	[211]	–
	Blautia	[206, 210, 211]	[122, 205]
Depression	Bifidobacterium	–	[121, 222, 232]
	Lactobacillus	–	[222]
	Faecalibacterium prausnitzii	–	[121, 231, 232]
	Blautia	[121]	–
	Escherichia	[232]	[121]
	Shigella	[232]	[121]
	Klebsiella	[121]	–
	Coprococcus	–	[96]
Autism spectrum disorder	Klebsiella	[123, 242]	–
	Clostiridium species	[123]	[246]
	Bifidobacterium	–	[123, 243]
	Escherichia	[245–247]	–

The associations between neurological disorders (Alzheimer's disease, Parkinson’s disease, multiple sclerosis, depression, and autism) and bacteria (genus or species). The sources of these associations range from the latest studies (in humans or rodents) to review papers. The PubMed query used was: ((Genus) OR (Species)) AND ((Alzheimer's disease) OR (Parkinson's disease) OR (Multiple sclerosis) OR (Depression) OR (Autism))

Glutamate is an excitatory neurotransmitter, and its synthesis by gut microbes is particularly unique in its metabolic pathway [119, 130]. This synthesis involves acetate production as a metabolite, a product of carbohydrate fermentation in the gut [119]. Bacteria such as *Bifidobacterium* generate acetate through decarboxylation of pyruvate to acetyl-CoA and afterward to acetate via acetyl-CoA hydrolase [131]. Acetate is considered a SCFA, and as mentioned earlier, it can increase 5-HT levels via upregulation of TpH1 [118]. Glutamate itself cannot permeate the BBB. However, acetate can cross the BBB and participate in glutamate-glutamine synthesis [119, 130]. Essential gut microbiota contributing to glutamate synthesis in the gut include *Lactobacillus plantarum*, *Bacteroides vulgatus*, and *Campylobacter jejuni* [132]. Elevated levels of glutamate have been associated with AD and PD. A proposed hypothesis suggests that the overexcitation of neurons can lead to neuronal death, promoting the pathogenesis [133, 134]. Interestingly, a recent study on mice found that administering a gavage rich with *Bacteroides thetaiotaomicron* was associated with decreased levels of circulating glutamate [135]. However, high levels of fecal *B. thetaiotaomicron* were associated with AD and depression in the antibiotics-induced mice model [136, 137]. Furthermore, SG has shown to reverse reduced abundance of *B. thetaiotaomicron* that’s associated with patients suffering from obesity [135]. Thus, we need to investigate the relationship between B. thetaiotaomicron and neurological disorders further. In Tables 1, 2, both post-bariatric surgery and neurodegenerative diseases are shown to be associated with lower *Lactobacillus* levels.

This same acetate can also synthesize Gamma-Aminobutyric Acid (GABA) as an inhibitory neurotransmitter. Although GABA cannot traverse the BBB, its precursor, acetate, can successfully cross. Within GABAergic neurons, glutamate is converted into GABA through the action of the enzyme glutamic acid decarboxylase. The synthesis of GABA in the gut involves *Bifidobacterium*, *Bacteroides fragilis*, *Parabacteroides*, *Eubacterium,* and *Lactobacillus* [119, 138]. *Lactobacillus* possesses an enzyme referred to as glutamate decarboxylase system, which converts L-glutamate to GABA [138]. This enzyme is also present in *Bifidobacterium* [139]. Interestingly, the addition of probiotics such as *Lactobacillus* and *Bifidobacterium* in both mice and humans has been shown to increase GABA levels in the gut and the brain, potentially explaining a direct link between these two systems [140]. Tables 1 and 2 demonstrate an association between a low GABA-producing microbiota profile, such as *Bifidobacterium* and *Lactobacillus*, in post-bariatric surgery patients and conditions like AD, MS, Depression & ASD but not PD gut-microbiota.

No concrete evidence currently indicates that RYGB surgery directly alters neurotransmitter levels in plasma. However, studies have shown an increase in fecal levels of 5-HT, glutamate, and GABA following surgery, suggesting that gut metabolism may be indirectly affected [141–143]. Evidence suggests that dopamine receptor levels may normalize post-RYGB, as obesity is often associated with altered dopamine signaling, including decreased receptor availability due to overstimulation of the reward system from high baseline dopamine activity. RYGB appears to restore these receptor levels to a normal state [144, 145].

Regarding GABA, specifically GABA-A receptors in the hypothalamus, studies on rat brains have shown that their numbers increase after surgery, independent of calorie intake [146]. Lastly, metabolic abnormalities in serum, including disruptions in glycine, serine, and threonine pathways, which are involved in the metabolism of some of the aforementioned neurotransmitters, were detected in post-RYGB candidates experiencing weight regain compared to those with sustained weight loss. These findings emphasize the role of metabolism changes in post-surgical weight outcomes [147].

### Influence of gut microbiota on neuroinflammation

A balanced, healthy microbiota is central to maintaining equilibrium between pro-inflammatory and anti-inflammatory responses. It achieves this balance by releasing anti-inflammatory cytokines, such as Interleukin-10 (IL-10), Transforming Growth Factor Beta (TGF-β), and Transforming Growth Factor Alpha (TGF-α), alongside pro-inflammatory cytokines like Interleukin-1β (IL-1β), Interleukin-17 (IL-17), Interferon-gamma (IFN-γ), and Tumor Necrosis Factor-Alpha (TNF-α) [88]. Conversely, an abnormal microbiota composition can disrupt this equilibrium, pushing it towards chronic inflammation and giving rise to autoimmune and inflammatory conditions [88]. Notably, research indicates that an aberrant microbiota composition can also precipitate neuroinflammation, leading to neurodegenerative and neuropsychiatric disorders [88].

Researchers posit that neuroinflammation may be triggered by increased permeability of both the BBB and the gut barrier [88, 89]. This increased permeability arises from changes in TJs expression between epithelial cells. TJ expression may be downregulated depending on the specific interactions involved, such as direct influence from gut microbe antigens or cytokines. This disruption in the integrity of TJs can lead to increased movement of substances between compartments and, consequently, promote inflammation [88, 115]. In addition to the TJs, the thickness of the mucous layer also acts as a protective barrier [148]. Certain gut microbiota strains support the maintenance of a healthy mucous layer, while others do not [149]. When the mucous layer is compromised, endothelial cells are exposed and vulnerable [148, 149]. This vulnerability facilitates the paracellular transport of microbes and metabolites, including bacterial endotoxin LPS [114]. The increased permeability of the gut barrier allows LPS to cross into the bloodstream, and subsequently, it may cross the BBB, potentially inducing neuroinflammation [114].

This suggests the importance of maintaining a healthy gut microbiota in preventing the deleterious effect of dysbiosis, which can target the brain through many pathways. Indeed, neuroinflammation is a dangerous consequence of gut dysbiosis, and chronicity can trigger a plethora of neurological disorders.

The effects of bariatric surgery on inflammation have been well-documented. Research shows that systemic inflammation markers, including pro-inflammatory cytokines (e.g., TNF-α, IL-6) and C-reactive protein (CRP), and abdominal fat density are significantly reduced within weeks to months after bariatric surgery [56, 150]. Additionally, studies on mice have demonstrated that RYGB can reverse neuroinflammation related to hypothalamic TLR4 signalling [151]. However, most studies focus on short-term outcomes, and further investigation is needed to understand the long-term consequences of the changes in microbiota profile, especially as neurodegenerative disorders typically develop over extended periods.

## Neurological conditions associated with altered gut microbiota

**Fig. 2:**
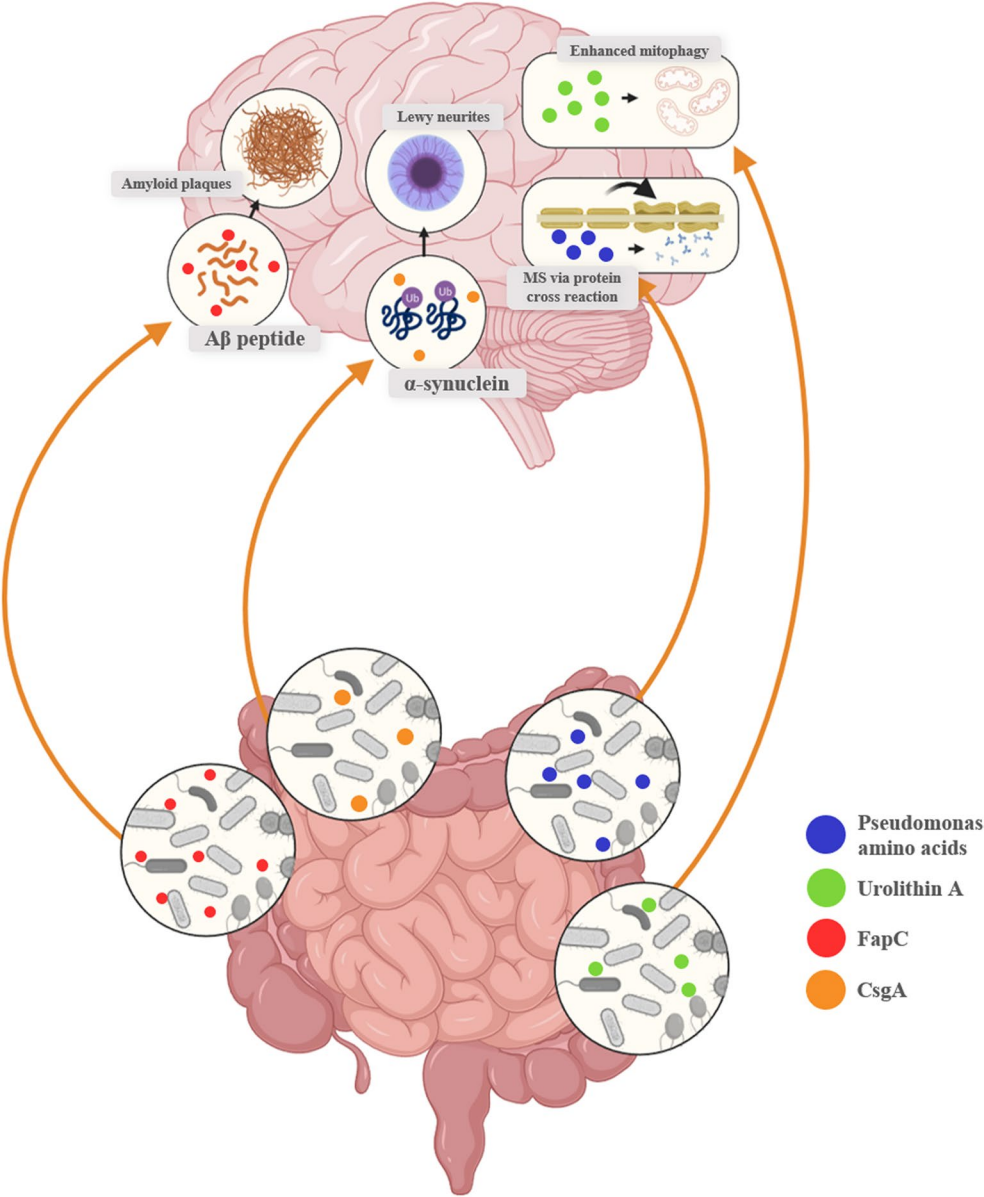
Schematic diagram illustrating the influence of microbial interactions on neurological disorders. Microbes and their metabolites have an ability to influence the CNS. For example, *Pseudomonas* has the ability to produce FapC, an amyloid fragment in the extracellular matrix, which can bind to Aβ, facilitating its deposition. In an inflamed gut, and consequently an inflamed BBB, FapC may promote aggregation in the brain, potentially contributing to AD [162]. Similarly, *Escherichia coli* metabolites, such as pili proteins and LPS, have been documented on amyloid plaques and may act via the same mechanism [160]. Moreover, Enterobacteriaceae have been shown to produce an amyloid protein called ‘Curli,’ which has a subunit referred to as CsgA that has been shown to promote alpha-synuclein aggregation, contributing to PD [185]. In addition, *Pseudomonas* amino acids have been shown to cross-react with myelin essential protein, leading to autoantibody formation against myelin and potentially development of MS [208]. Lastly, *Adlercreutzia* has been shown to protect against AD via a mitochondrial metabolite called Urolithin A, promoting anti-inflammatory actions and enhancing mitophagy, which is a risk factor for AD if impaired [174]. *AD *Alzheimer’s disease, *BBB* Blood brain barrier, *LPS* Lipopolysacchride, *PD* Parkinson’s Disease, *MS* Multiple sclerosis

Neurodegenerative diseases such as AD, PD, and MS are increasingly causing morbidity worldwide [152, 153]. This morbidity extends to mental and developmental disorders such as Depression and ASD. This section explores each neurological disorder and its association with bariatric surgery and gut microbiota. Unfortunately, there have been minimal clinical or animal studies that focused on researching any association between post-bariatric surgery, gut dysbiosis, and neurological disorders. Nevertheless, multiple observational studies conducted post-bariatric surgery in the context of obesity have provided an opportunity to indirectly correlate the general microbiota composition observed after bariatric surgery, as shown in Table 1, with the gut microbiota composition associated with neurological disorders, as shown in Table 2. This correlation might aid in establishing a possible connection linking gut microbiota, post-bariatric surgery, and neurological disorders. Moreover, Fig. 2 presents a schematic diagram illustrating the potential consequences of select microbial metabolites associated with neurodegenerative disorders, which are explained in detail in the upcoming sections.

### Alzheimer’s disease

AD is the most common cause of dementia worldwide, affecting more than 44 million individuals globally [154]. The pathogenesis of AD includes deposition of extracellular amyloid beta (Aβ) and abnormal tau protein hyperphosphorylation, leading to the formation of extracellular neurofibrillary tangles, all of which contribute to the loss of synapses and eventually leading to neuronal death [155, 156]. A study on mice has shown that peripheral beta-amyloid, especially the one produced from the gut microbiota, might play a crucial role in AD pathogenesis [157]. Additionally, the mechanisms of how gut microbiota dysbiosis can contribute to AD are complex and include various pathways such as neuroinflammation, immune modulation, and direct gut-brain communication via the vagus nerve [158].

Multiple studies have shown that the population of various *Pseudomonas* species was found to be increased in AD patients [159–161]. This bacterium has been associated with the pathogenesis of AD through the ability to promote Aβ deposition, the hallmark protein of AD, possibly through the production of FapC, an amyloid fragment in the extracellular matrix of the bacteria that binds to Aβ facilitating its deposition [162]. *Escherichia* and *Shigella* were other pro-inflammatory bacteria whose levels were found to be also increased in patients with AD [163–166]. Metabolites from *Escherichia*, such as pili protein and LPS, were present in amyloid plaques of post-mortem AD patients, giving a strong indication of how *Escherichia* might be involved in the pathogenesis of AD [160]. Another gut bacterium positively correlated with AD was *B. thetaiotaomicron* [136]. The probable pathogenesis behind the bacterium is that since it’s a gram-negative bacterium, it possesses LPS, which induces inflammation, potentially resulting in neuroinflammation [136].

Additionally, *B. thetaiotaomicron* has been shown to bind to polysaccharides in the gut through their unique pili. As a result, it does not allow other bacterial species to utilize this energy resource [136]. Thus, it could alter the gut microbial population and even reduce anti-inflammatory populations dependent on polysaccharides such as *Bifidobacterium* [167]. This sheds light on the direct involvement of certain bacteria in promoting the pathogenesis of AD, with some possessing amyloid proteins that promote Aβ deposition. Meanwhile, others carry increased risk factors for AD, such as elevated levels of pro-inflammatory metabolites.

On the other hand, specific bacteria were shown to have protective factors. *Bifidobacterium* is a well-known anti-inflammatory bacterium that was shown to be generally decreased in AD patients [168–170]. This bacterium is known to be negatively correlated with LPS concentration, involved in maintaining gut integrity, and possesses an anti-inflammatory effect by preventing the translocation of specific endotoxins [171]. These are all protective factors for AD and, in fact, for the majority of neurological disorders. Moreover, rodent studies also showed a reduction in Aβ deposition when *Bifidobacterium*-containing probiotics were administered to them [172].

Similarly, *Lactobacillus* is considered a vital probiotic and has been shown to have a protective effect through the production of SCFA, which is known for its anti-inflammatory properties. Moreover, rodent studies have shown that *Lactobacillus* probiotics reduced amyloid plaque deposition, similar to *Bifidobacterium* [173]. *Lactobacillus* population was also reported to be decreased in fecal samples of AD patients [170]. *Adlercreutzia* is another notable bacterium that has been shown to protect against AD by producing a beneficial mitochondrial metabolite called Urolithin A, which has anti-inflammatory properties and enhances mitophagy – the process of removal of damaged mitochondria– which, if impaired, is considered a risk factor for AD [174]. *Coprococcus* was shown to have a protective effect as it promotes anti-inflammatory properties by producing SCFA. Like the above bacteria, low levels of gut *Coprococcus* species were associated with certain features of AD [163]. Lastly, *Akkermansia* is known to be protective in the case of AD and is involved in maintaining the integrity of the gut barrier by repairing damaged epithelial cells [175]. In mice AD models, it was found that the population of gut *Akkermansia* for AD was decreased as well [176]. This shows that some bacteria have potential protective factors against AD by promoting anti-inflammatory effects, which explains how low levels of these bacteria can potentially promote AD.

Bariatric surgeries for weight loss are known to disrupt gut bacterial populations and composition widely. Proinflammatory bacteria such as *Pseudomonas*, *Escherichia*, and *Shigella* were all found to be increased after post-bariatric surgery [29, 30]. Moreover, anti-inflammatory bacteria were found to be generally decreased after surgical weight loss interventions. These included the ones mentioned above, such as *Bifidobacterium*, *Lactobacillus*, and *Coprococcus* [27, 29, 30]. For other anti-inflammatory bacteria, namely *Akkermansia*, it was found that the population actually increased post-RYGB surgery [29].

To sum up, the gut microbiota of AD patients and individuals who underwent post-bariatric surgery share some overlapping associations. However, these observations do not entirely elucidate the situation as a whole, and it is always best to keep in mind that the gut microbiome is a complex environment in which one small change can lead to rapid magnified changes down the line. One notable non-overlapping association was with *Akkermansia*; its increase in population post-bariatric surgery exhibits protective factors against AD. This might be due to some compensatory mechanism or complex interactions yet to be studied. If these associations were found to be very significant in the future, the benefit-to-risk ratio should always be considered, especially for patients with a family history of AD. Additionally, alternative treatment options can be considered, or the addition of probiotics after surgery could also be beneficial.

### Parkinson’s disease

PD is the second most common cause of neurodegenerative disease worldwide, affecting more than 8.5 million people globally [177]. It is characterized mainly by motor symptoms such as rigidity, tremors, and bradykinesia. Other symptoms include depression, sleep disturbances, and constipation [178]. The pathogenesis of PD is complex and involves multiple factors. It includes inflammatory processes and neurodegeneration, primarily in dopaminergic and some nondopaminergic brain areas. Additionally, the disease is characterized by Lewy neurites and bodies forming, which are made from deposits of α-synuclein protein. This protein is widely considered the center of PD pathogenesis [179].

Research has shown that dysbiosis, leading to increased proinflammatory bacterial growth, can contribute to inflammation-induced misfolding of α-Synuclein [180]. Indeed, proinflammatory gut bacteria were found to be generally increased in PD patients and include species from Proteobacteria and Enterobacteriaceae [180–183]. For instance, Enterobacteriaceae has been positively associated with abnormal postural and gait symptoms of parkinsonism [183, 184]. The proposed pathogenesis involves the production of a bacterial amyloid protein called 'Curli,' which consists of a significant structural subunit called CsgA. This subunit promotes α-synuclein aggregation and induces motor abnormalities in mice overexpressing α-synuclein [185]. In addition, *Klebsiella* was shown to promote amyloid fibril formation in cell lines, a hallmark for both PD & AD [186]. Moreover, its fecal abundance in PD patients was shown to correlate with the severity of the disease alongside another bacteria, *Parasutterella* [187].

Anti-inflammatory SCFA-producing bacteria in fecal samples and mucosal sigmoid biopsies were found to be reduced in PD patients, including species from *Coprococcus*, *Blautia*, and *Roseburia* [180]. Other studies have shown that decreased *Blautia* accelerated PD [188]. *Bifidobacterium* is a bacterial genus that is well known to confer a neuroprotective role due to it being SCFA-producing, contributing to the reduction of α-synuclein aggregation and slowing down the loss of dopaminergic neurons [184]. Interestingly, fecal levels of *Bifidobacterium* were found to be generally elevated in PD patients [189]. A plausible explanation for this, as mentioned in the AD section, is that it may represent the body’s attempt to compensate for neurodegeneration and the progressing pathology. However, the exact mechanism underlying this paradoxical increase remains to be investigated.

Another SCFA-producing bacterium, *Lactobacillus*, has shown mixed data associations in PD [190]. It is suspected that PD patients using COMT inhibitors, particularly, as part of their treatment increased both *Bifidobacterium* and *Lactobacillus* [190]. To further solidify *Lactobacillus’s* benefit in PD pathogenesis, studies have demonstrated that *Lactobacillus* can slow the progression of the disease by reducing microglial reactivity and regulating oxidative damage [191]. Furthermore, rodent experiments have indicated that these bacteria are associated with increased metabolite taurine, which has been shown to confer neuroprotective effects [191]. L-dopa is one of the main treatments for PD, and its effective dose fluctuates based on the gut microbiota. A meta-analysis concluded that *H. pylori* might be associated with the risk of developing PD, while other studies have shown that *H. pylori* can cause decreased and fluctuating L-dopa absorption in PD patients [192, 193]. In addition, *Enterococcus faecalis* possesses the enzyme tyrosine decarboxylase, which has also been shown to diminish L-dopa bioavailability in the gut [194]. Oddly, patients treated with L-Dopa have shown an increased dosage requirement corresponding with higher *Bifidobacterium* and *Clostridium* species [195, 196].

Post-bariatric surgery, namely RYGB, can cause gut microbiota dysbiosis that could be involved in worsening or improving PD symptoms. Bacteria that are thought to be cellulose-breaking, like *Blautia*, *Ruminococcus*, and *Faecalibacterium* were shown to be significantly decreased in PD patients compared to control. Moreover, they were negatively correlated with disease severity and duration [197]. Interestingly, certain studies have shown that *Blautia* tends to increase following bariatric surgery, particularly vertical SG [28, 188]. However, other studies suggest a decrease in *Blautia* populations post-bariatric surgery, as illustrated in Table 1 [30]. The discrepancies may arise from different populations being analyzed in these studies, leading to conflicting results. On the other hand, many bacteria species from the phylum Gammaproteobacteria were increased in PD patients, including *Klebsiella*, *Salmonella*, and *Shigella* [198, 199]. A similar positive correlation between RYGB and Gammaproteobacteria was observed in rodents after 9 weeks when compared to the sham surgery, a placebo-controlled surgical group. However, SG displayed changes similar to RYGB only during the first week [32]. Additionally, a study on human fecal samples confirmed elevated levels of Gammaproteobacteria following RYGB [33]. Similar to AD, *Akkermansia* plays an unclear mechanistic role in PD. However, its levels were seen to be positively correlated in PD patients [200]. Bariatric surgeries such as RYGB showed increased *Akkermansia*, and this association was even more pronounced in vertical SG surgery [28, 201]. For *Bifidobacterium*, a review summarizing the results of 14 clinical studies showed that bariatric surgeries tend to decrease the population of the probiotic neuroprotective bacteria [29].

Overall, in patients with PD, proinflammatory gut microbiota promoting α-Synuclein misfolding were found to be increased, while anti-inflammatory bacteria were found to be generally decreased. Moreover, certain dysbiosis would result in more severe PD symptoms while at the same time making the disease more challenging to treat. Furthermore, findings show that gut dysbiosis due to bariatric surgery could worsen or improve PD symptoms by affecting the intricate gut-brain axis. These findings also seem to suggest that if bariatric surgery is needed for a PD patient or a patient susceptible to developing PD due to family history, vertical SG seems to generally be a more beneficial option from a gut dysbiosis point of view as seen in Table 1.

### Multiple sclerosis

MS is an autoimmune disease characterized mainly by chronic inflammatory destruction of myelinated axons in the CNS, affecting an estimated 2.8 million people worldwide, with the prevalence only projected to increase with time [202, 203]. While the exact cause of developing MS remains unclear, it is believed to involve a combination of genetic susceptibility and environmental triggers. Other studies even suggest the involvement of the gut microbiota [204, 205].

A systematic review concluded that patients with MS are more likely to have less SCFA-producing bacteria, which already gives a reasonable explanation for chronic inflammation. The study concluded that there was an increase in proinflammatory bacteria such as Bacteroidetes and *Ruminococcus*. In contrast, a decrease in SCFA-producing bacteria such as Firmicutes*, Prevotella*, *Faecalibacterium prausnitzii*, *Bifidobacterium*, *Coprococcus*, *Lachnospiraceae*, *Butyricicoccus*, and others was also observed [206]. However, for *Akkermansia*, particularly *A. muciniphila*, more recent studies suggest that this elevation is believed to take part in a protective compensatory factor in MS patients, with results showing a positive correlation with brain volume and a negative correlation with lesion sizes and general disability symptoms [122, 200]. Further analysis to investigate a possible explanatory mechanism had shown a negative correlation between the *Akkermansia* gut population and RORγT + and IL-17 producing T-cells, a transcription factor and a cytokine, respectively, both thought to play a central role in the progression and severity of autoimmune diseases like MS [122]. In the case of gastric bypass surgeries, an apparent increase in *Akkermansia* abundance is a commonly reported outcome, likely driven by increased GLP-1 secretion, which alters glucose metabolism and, consequently, the intestinal flora [29, 207]. *Pseudomonas* was associated with MS, as seen in Table 1, and its amino acid can cross-react with myelin essential protein forming antibodies, possibly explaining the pathogenesis of MS, which could be of a similar mechanism to the molecular mimicry etiology seen in other conditions such as rheumatic fever [208].

*Blautia* is a known bacterium generally considered anti-inflammatory and protective [209]. However, the relative abundance of *Blautia* in MS patients is not entirely clear. Certain studies have shown that its population was decreased in MS patients [122, 205]. On the other hand, other studies have shown contrasting results, and an increased population [210, 211]. A systematic review of 12 studies has also highlighted this inconsistency [206]. A possible explanation for this discrepancy could be related to the type of immunomodulatory medications used in MS treatment, the disease stage and type, or other uncontrolled factors, such as the demographics and age of the study participants. It is possible that *Blautia* is not directly related to MS, and the observed changes may be secondary. Moreover, even though *Blautia* is generally deemed to be anti-inflammatory, it has been linked both negatively and positively to inflammatory conditions [210]. Regardless, the association between the *Blautia* population and bariatric surgery is less vague, in which generally, the species of the anti-inflammatory bacteria was shown to be decreased after the surgery was done [212]. However, as mentioned above, other studies suggest an increase in the *Blautia* population, especially post-vertical SG [28].

Obesity has been linked to the development of several autoimmune diseases, like MS, through the modulation of pro- and anti-inflammatory adipokines [212]. Therefore, bariatric surgery might be initially considered a good option to reduce the risk of developing MS by treating the risk factor of obesity. However, considering the above discussions about gut dysbiosis, it is essential to evaluate the actual risks and conduct further research to determine whether weight loss surgery is beneficial in decreasing the chances of developing MS, especially in patients with an extensive family history.

### Depression

Depression or major depressive disorder (MDD) is a severe psychiatric condition characterized by a persistent feeling of sadness and loss of interest in doing daily life activities [213, 214]. The prevalence of MDD in older people has been reported to be around 13.3% globally. At the same time, it is much higher in adolescents, with 34% of adolescents worldwide at risk of developing clinical depression [215, 216]. Like neurodegenerative diseases, the pathophysiology of MDD is complex and has been associated with genetic and various environmental factors, including the gut microbiota [217, 218].

As mentioned in previous sections, the gut microbiota is heavily involved in synthesizing neurotransmitters, especially ones important in mood regulation, such as GABA, dopamine, norepinephrine, 5-HT, and others [90]. Therefore, any alteration to the gut microbiome can disrupt neurotransmitter homeostasis, potentially contributing to mood disorders and neurodegenerative conditions [90, 119]. GABA is a CNS inhibitory neurotransmitter that has been shown to play a role in both depression and anxiety [219]. *Bacteroides fragilis*, certain *Bifidobacterium*, and *Lactobacillus* species were found to be some of the most prominent bacteria in the gut microbiome that can produce GABA, while the recently discovered *Evtepia glamorous* (KLE1738) is considered a “GABA-eating” species [220, 221]. In the case of depression, both *Bifidobacterium* and *Lactobacillus* levels in fecal samples were found to be decreased in MDD patients [222]. Comparing this to bariatric surgery, RYGB was shown to increase the *Bacteroides* population while decreasing the *Lactobacillus* population, as seen in Table 1 [29, 223]. Another neurotransmitter that is widely associated with mood disorders such as depression is 5-HT and its production can be affected by gut microbiota as discussed earlier [224].

Both depression and anxiety were linked to neuroinflammation and an unhealthy gut microbiome [225–227]. For example, it was shown that inflammatory cytokines such as IL-6, IL-8, and TNF-α were increased in the blood and CSF of patients suffering from MDD [228–230]. Patients with MDD showed an increase in proinflammatory bacteria such as *Klebsiella*, and *Desulfovibrio* while at the same time showing decreased levels in the anti-inflammatory SCFA-producing bacteria, namely *Faecalibacterium* and *Bifidobacterium* [121, 231, 232]. However, it’s not clear for some pro-inflammatory bacteria, such as *Escherichia* and *Shigella* as they are reported differently [121, 232]. Nonetheless, probiotics containing *Bifidobacterium* and *Lactobacillus* were effective in treating depression symptoms [233]. For gastric bypass, results report an increase in *Bifidobacterium* post-surgery along with other species related to MDD, such as *Klebsiella* [234]. However, as listed in Table 1, *Bifidobacterium* has shown a reduction in post-bariatric surgery.

Overall, the associations between MDD and various gut bacteria are vast but present. Two main mechanisms that link the gut microbiota to MDD are neurotransmitter production and inflammation, among others. The association between microbiota changes and bariatric surgery further prompts the use of probiotics containing *Lactobacillus* and *Bifidobacterium* to mitigate symptoms that commonly occur, such as depression, especially with people predisposed to it, such as a past medical history or family history of MDD [233, 235].

### Autism spectrum disorder

ASD is a neurodevelopmental disorder characterized by restricted interest and repetitive behavior [236]. Around 1 in every 100 children is diagnosed with ASD worldwide [237]. ASD has been associated with many factors, including genetic and environmental factors, and, more recently, gut dysbiosis [238, 239]. The relationship between ASD and the gut-brain axis is being extensively researched, so much so that fecal transplants containing *Bifidobacterium*, *Streptococci*, and *Lactobacillus* are becoming increasingly promising therapeutic approaches [240, 241].

Some bacteria that have been widely considered to be positively correlated with ASD are *Klebsiella* and *Clostridium* species [123, 242]. Similar to the diseases discussed above, *Bifidobacterium* was found to be decreased in patients with ASD [243]. The pathogenesis implicated here seems to be more associated with intestinal GABA production, as patients with a lower likelihood of developing ASD had lower fecal GABA than patients with an increased likelihood of developing ASD [123]. Moreover, in vitro studies confirmed that *Clostridium* species consume GABA while *Bifidobacterium* secretes GABA, as mentioned above in the depression section [123]. Additionally, ASD patients were found to have altered tryptophan metabolism with low tryptophan levels and high 5-HT levels in the blood [117, 240, 244]. Therefore, the organisms mentioned in the depression section, namely *Escherichia*, could play a role in ASD pathogenesis via these 5-HT alteration mechanisms. Finally, another prominent bacterium that seems to be implicated in ASD is Escherichia. A recent study reported an increase in Escherichia levels in children with ASD, suggesting that, similar to Klebsiella, its pro-inflammatory properties might contribute to the pathogenesis of ASD [245]. However, an older study using the same fecal analysis method and conducted on a similar demographic reported a decrease in Escherichia levels [246]. Notably, the sample size in the older study was less than half that of the more recent study. This discrepancy is challenging to explain given the similarities in methodology and population, but it is most likely attributable to the larger sample size of the more recent study, which makes its findings more reliable. Moreover, an even more recent comparative study examining children with ASD and their siblings further supports an increase in the Gammaproteobacteria population, which encompasses Escherichia [247].

Along with gut dysbiosis, many patients with ASD have gut abnormalities such as increased gut permeability and infections [248]. The increased gut permeability, in particular, can contribute to further gut dysbiosis, leading to lower beneficial *Lactobacillus* strains and possibly further pathogenesis [249]. However, treating MIA rodents, a model for ASD, with *Bacteroides fragilis* probiotics caused an improvement in gut permeability treating it along with some ASD symptoms [244].

As mentioned in other sections, weight loss surgeries lead to a varying degree of dysbiosis. Species such as *Klebsiella* were found to be increased post-surgery, while GABA-producing *Bifidobacterium* species were found to be decreased [29]. Both of these overlap with the associations found in ASD. However, for GABA-consuming *Clostridium* species, the overlap remains to be seen as its abundance increased after RYGB but decreased after SG [30]. Taking all of this into consideration, children with obesity and ASD should be evaluated on a case-by-case basis, as more and more evidence has been supporting a deep link between the gut-brain axis and the development of ASD [250].

## Therapeutic implications and future directions

The growing prevalence of neurological conditions necessitates the development of effective treatments. While research indicates a positive link between bariatric surgery, microbiota, and cognition, challenges persist. Many studies on gut microbiota and neurological conditions rely on animal models, limiting their applicability to humans. Furthermore, many studies are correlational rather than causal, necessitating more randomized controlled trials and prospective studies. Long-term effects of bariatric surgery are rarely reported, and multiple confounding variables, such as diet and demographics, complicate gut microbiota studies.

Building on this understanding, researchers have explored therapeutic interventions for weight loss. General associations between weight loss treatments and health improvements have been identified, but limitations in data and variability in individual responses still need to be improved. Ozempic (semaglutide), initially developed for diabetes management, has shown significant potential for weight loss, although the data on its long-term associations and effects are still limited [251, 252]. Additionally, tirzepatide, the first dual GIP /GLP-1 receptor co-agonist approved for treating T2DM in several regions, has demonstrated unprecedented reductions in both HbA1c and body weight [253]. GLP-1 agonists, such as semaglutide, may also alleviate depression symptoms in patients with diabetes, possibly through their anti-inflammatory effects and the neuroprotective potential of GLP-1 [254, 255]. In addition, semaglutide was also found to improve the integrity of the gut barrier and reverse dysbiosis. For example, research on the effects of semaglutide in obese mice showed that the treatment not only improved cognition and reduced inflammation but also significantly modified the gut microbiota. Specifically, semaglutide reversed the dysbiosis associated with a high-fat diet, with a notable increase in Akkermansia levels. This finding underscores its potential role in mitigating the detrimental effects of such diets [256]. Moreover, a similar study has that shown dysbiosis was reversed in obese mice models treated with semaglutide, leading to an increase in Akkermansia and *Faecalibaculum* and a decrease in *Lactobacillus* and *Bacteroides* [257]. While these changes do not seem to be particularly pushing towards anti- or pro-inflammatory bacteria, GLP-1 agonists were shown to have an overall anti-inflammatory response and the implications to treat a variety of diseases other than T2DM including inflammatory bowel disease (IBD) and neurodegenerative disease are being widely studied [258]. For example, administration of semaglutide was shown to decrease both α-Synuclein and Aβ plaque deposition in animal models making them potentially beneficial for the management of obesity in patients with PD or AD [259–261]. Interestingly as well, semaglutide was found to be beneficial in attenuating MS and promoting remyelination in mice models [262]. All of this demonstrates how GLP-1 agonists could be used in the future to treat obesity and also provide a degree of neuroprotection.

Other interventions targeting gut microbiota include probiotic supplements and FMT. Probiotics have shown significant reductions in ASD severity and improvements in PD symptoms [263, 264]. A recent study found a significant reduction in the severity of ASD following probiotic and fructo-oligosaccharide administration [263]. In this study, analysis of the gut microbiota profiles revealed that the gut microbiota of children with ASD was in a state of dysbiosis, with significantly lower levels of *Bifidobacterium longum* and *Bifiobacteriales* at baseline compared to neurotypical children. However, after probiotic and fructo-oligosaccharide administration, *Bifidobacterium* levels increased, accompanied by a significant reduction in autism severity [263]. These results suggest that a change in *Bifidobacterium* levels following probiotic treatment is correlated with ASD severity, highlighting the potential of microbiota-based approaches in the treatment of ASD. Since bariatric surgery induces gut microbiota alterations, it prompts consideration of its potential as a treatment option for ASD.

Another study examined the impact of probiotic consumption by individuals with PD, using the Movement Disorders Society-Unified Parkinson’s Disease Rating Scale (MDS-UPDRS). Compared to the placebo group, the study revealed that a 12-week regimen of probiotic intake led to a significant reduction in MDS-UPDRS scores [265]. Probiotic use was also associated with lower levels of high-sensitivity CRP and malondialdehyde, while enhancing glutathione levels compared to the placebo group. Moreover, probiotic intake significantly lowered insulin levels and improved insulin sensitivity [265].

FMT involves the transfer of healthy microbiota to afflicted individuals and has been shown to reduce neuroinflammation and improve symptoms of neurodegeneration [264]. In a study using a transgenic mouse model of AD (ADLP^APT^) that replicates key AD pathological features, researchers explored the role of gut microbiota in AD pathogenesis [266]. Frequent fecal microbiota transfers from healthy wild-type mice to ADLP^APT^ mice resulted in a significant reduction in amyloid β plaques, neurofibrillary tangles, glial reactivity, and cognitive impairment. [266]. The findings suggest a link between microbiota-mediated immune aberrations in the gut and systemic inflammation contributing to AD pathogenesis. They also highlight the potential therapeutic approach of restoring gut microbial homeostasis for treating AD.

In a recent study, a chronic rotenone-induced PD mouse model was used to investigate the impact of gut microbiota dysbiosis on PD pathogenesis through the microbiota-gut-brain axis [267]. Rotenone administration induced gut dysbiosis, leading to gastrointestinal dysfunction and behavioral deficits in PD mice. FMT treatment was shown to effectively restore the gut microbial community, improving gastrointestinal functions and motor deficits. The study further revealed that FMT reduced intestinal inflammation, preserved blood–brain barrier integrity, and suppressed neuroinflammation in the substantia nigra, thereby protecting dopaminergic neurons. Mechanistically, FMT lowered LPS levels and inhibited the TLR4/NF-κB signaling pathway in the colon, serum, and substantia nigra. These findings suggest that FMT mitigates PD by addressing gut microbiota dysbiosis and its downstream inflammatory pathways, emphasizing its potential as a therapeutic strategy for PD [267].

Furthermore, a recent comprehensive review has demonstrated that some FMT studies in humans have generally shown improvement in motor symptoms of MS and PD, while other studies yielded mixed results regarding symptomatic improvements in MS [268]. The review also reported cognitive improvements in patients with PD and dementia [268]. Additionally, a randomized, double-blind placebo-controlled pilot study involving 12 participants has demonstrated that FMT improved both motor and non-motor symptoms in patients with PD [269]. While these interventions show promise, further clinical trials involving probiotic supplements and FMT are needed to ensure their safety and efficacy.

## Conclusion

The bidirectional gut-brain axis exerts a profound effect on the central nervous system. The gut, housing microbiota, can directly influence the CNS, contributing to various disorders, including neurological conditions. While developing the various neurological conditions mentioned in this review is definitely multifactorial, nevertheless, the interplay among gut microbiota, bariatric surgery, and neurological studies still needs to be explored. This review elucidates the general association between these three major domains. We aim to establish connections between bariatric surgery, microbiota composition, and neurological disorders, forming a triad. Overall, bariatric surgery was shown to significantly modulate the normal gut flora. While some anti-inflammatory bacteria were shown to be increased, there is also an observed rise in harmful bacteria, such as *Escherichia* and *Clostridium*, which were shown to be associated with various neurological disorders. This revelation sheds new light on the potential implications of post-bariatric surgery microbiota composition in the context of neurological disorders. We anticipate our review will pave the way for more sophisticated experimental studies exploring the triad of gut microbiota, bariatric surgery, and neurological disorders. Furthermore, we propose investigating methods to address potential complications of bariatric surgery, such as using probiotics and fecal microbiota transplants, to modulate or replace the microbiota residing in the gut, which may be a factor in inducing neurological disorders.

## Data Availability

All data generated or analyzed during this study are included in this published article.

## Abbreviations

5-HT: Serotonin
5-HTP: 5-Hydroxytryptophan
Aβ: Amyloid beta
ACh: Acetylcholine
AD: Alzheimer disease
ASD: Autism spectrum disorder
BBB: Blood–brain barrier
BMI: Body mass index
CNS: Central nervous system
CRP: C-reactive protein
FMT: Fecal microbiota transplant
FSH: Follicle-stimulating hormone
GABA: Gamma-aminobutyric acid
GIP: Glucose-dependent insulinotropic polypeptide
GLP-1: Glucagon-like peptide 1
HPA: Hypothalamus pituitary adrenal
HPG: Hypothalamus pituitary gonad
IFN: Interferon
IL: Interleukin
LH: Luteinizing hormone
LPS: Lipopolysaccharide
MDD: Major depressive disorder
MDS-UPDRS: Movement Disorders Society-Unified Parkinson's Disease Rating Scale
MS: Multiple sclerosis
PCOS: Polycystic ovary syndrome
PD: Parkinsons disease
PYY: Peptide YY
RYGB: Roux-en-Y gastric bypass
SadA: Staphylococcal aromatic amino acid decarboxylase
SCFA: Short-chain fatty acids
SG: Sleeve gastrectomy
T2DM: Type 2 diabetes mellitus
TGF: Transforming growth factor
TGR5: Takeda G protein-coupled receptor 5
TJ: Tight junction
TLR: Toll-like receptors
TNF: Tumor necrosis factor
TpH: Trp hydroxylase
TUDCA: Tauroursodeoxycholic acid
